# The role of spatial structure in the evolution of viral innate immunity evasion: A diffusion-reaction cellular automaton model

**DOI:** 10.1371/journal.pcbi.1007656

**Published:** 2020-02-10

**Authors:** Ernesto Segredo-Otero, Rafael Sanjuán

**Affiliations:** Institute for Integrative Systems Biology (I2SysBio), Consejo Superior de Investigaciones Científicas-Universitat de València, València, Spain; ETH Zurich, SWITZERLAND

## Abstract

Most viruses have evolved strategies for preventing interferon (IFN) secretion and evading innate immunity. Recent work has shown that viral shutdown of IFN secretion can be viewed as a social trait, since the ability of a given virus to evade IFN-mediated immunity depends on the phenotype of neighbor viruses. Following this idea, we investigate the role of spatial structure in the evolution of innate immunity evasion. For this, we model IFN signaling and viral spread using a spatially explicit approximation that combines a diffusion-reaction model and cellular automaton. Our results indicate that the benefits of preventing IFN secretion for a virus are strongly determined by spatial structure through paracrine IFN signaling. Therefore, innate immunity evasion can evolve as a cooperative or even altruistic trait based on indirect fitness effects that IFN shutdown exerts on other members of the viral population. We identify key factors determining whether evasion from IFN-mediated immunity should evolve, such as population bottlenecks occurring during viral transmission, the relative speed of cellular infection and IFN secretion, and the diffusion properties of the medium.

## Author summary

Innate immunity signaled by interferons is an important defense system against pathogens. Thus, it is often assumed that most pathogens can rapidly evolve strategies to avoid immune recognition or block interferon signaling. However, the presence of some interferon-stimulating pathogen variants in a given host may be sufficient for triggering innate immune responses, negatively impacting the fitness of interferon-blocking pathogens and posing a major hurdle for the evolution of innate immunity evasion. Here, we simulate the spread of viral infections and interferon-mediated innate immune responses in cell populations. We find that, by allowing for the insulation of interferon-stimulating and interferon-blocking virus variants, spatial structure should play a central role in the evolution of innate immunity evasion. In turn, we identify features determining spatial structure, such as the number of founder viruses, infection dynamics, medium viscosity, and virion size. Our results shed light on the evolution of a key process involved in pathogenesis and virulence.

## Introduction

Innate immune signaling during early infection constitutes an important line of defense against viruses. Infected cells recognize pathogen-associated molecular patterns and secrete type-I interferons (IFNs), which act in autocrine and paracrine manners to halt the spread of the infection locally [1, 2]. Autocrine signaling triggers antiviral responses in the secretor cell including gene expression arrest and apoptosis, whereas paracrine signaling induces a virus-resistant state in neighbor cells. Innate immunity imposes a strong selective pressure on viruses, which have consequently evolved a variety of evasion mechanisms at the level of viral sensing, signal transduction, and/or gene expression [2–4]. If successful, these mechanisms lead to avoidance or shutdown of IFN secretion.

We have recently shown that a mutant of vesicular stomatitis virus (VSV) that fails to block IFN secretion reduces the fitness of neighbor, IFN-blocking VSV by triggering local antiviral responses [5]. This interference defines IFN shutdown as a social trait, since the fitness of a given virus infecting a given cell (focal virus) depends on other members of the viral population (neighborhood). A neighborhood constituted by viruses that block or do not stimulate IFN provides a fitness advantage relative to being located near IFN-stimulating viruses. This fitness effect is indirect because it is determined by cells not infected by the focal virus, is exerted through paracrine signaling, and depends on the spatial distribution of infected cells and immunized cells.

Blocking IFN should also have a direct effect on the focal virus determined by autocrine signaling. In principle, since autocrine signaling is aimed at reducing viral progeny production, IFN blockade should directly benefit the focal virus. However, if the viral infection cycle proceeds faster than autocrine antiviral responses, this direct benefit may not be realized. In addition, directing viral proteins to prevent IFN secretion (by avoiding recognition or blocking production) might have a direct cost for the focal virus. For instance, in VSV, the matrix protein M prevents IFN secretion by inhibiting host gene expression [6], but M is also an essential structural component of the virion. Hence, using M proteins for blocking gene expression in the nucleus might be costly for virion morphogenesis. Another potential source of direct costs to IFN blockade is that anti-IFN viral proteins could downregulate cellular gene expression or trigger premature apoptosis, reducing the availability of cellular resources for viral progeny production. The existence of such costs is suggested by our previous results with VSV [5].

Therefore, IFN shutdown should have direct and indirect effects on viral fitness. Because indirect effects depend on other members of the viral and cellular populations, tackling the evolution of innate immunity evasion requires considering the spatial structure and the dynamics of viral spread and IFN-mediated responses. In general, the term spatial structure refers to a non-random arrangement of individuals in space, and can originate from physical barriers, limited dispersal, a tendency to aggregate, demographical history, and other processes related to population dynamics [7]. For instance, in viral infections, host-to-host transmission and intra-host dissemination is limited by anatomical barriers. Yet, even in the absence of such barriers, most viruses infecting solid tissues exhibit spatial structure, as evidenced by the formation of infection foci. Such foci can result from a simple diffusion-reaction process involving adsorption of viral particles to cells, production of viral progeny, and diffusion of progeny particles in the medium before reaching a new cell [8–11]. Analogously, the spatial structure of immune responses mediated by IFN and other cytokines can be determined by the diffusion of signaling molecules and the effect of these signals in receptor cells [12]. In addition to infection and immunity, diffusion-reaction models have been used for addressing a variety of eco-evolutionary questions such as, for instance, how bacterial CRISPR limits viral spread [13] and how spatial obstacles perturb population expansion fronts and promote random genetic drift [14].

Another useful approach to investigating viral spread is provided by cell automaton models, in which individual cells are simulated as elements of a grid, making spatial structure explicit [15–21]. This approach has been previously used for studying how the interaction between viruses and the immune system is influenced by space. For instance, it has been shown that spatial clustering of infected cells (which is a consequence of viral spread in foci) tends to reduce the ability of cytotoxic T-lymphocytes (CTLs) to clear the infection, but that clustering actually increases clearing efficacy if chemotaxis allows CTLs to perform a non-random search for infected cells [22]. Another work explored how spatial structure influences the evolution of CTL escape mutants in HIV-1 [23]. CTL escape mutant should be able to avoid CTL-mediated lysis regardless of the presence of non-mutant viruses in the same host because CTL-mediated lysis is epitope-specific. Nevertheless, CTLs can also secrete cytokines capable of repressing infection in an epitope-independent manner, for instance by reducing the overall susceptibility of cells to viral infection. CTL escape mutants might be sensitive to this mode of action and, consequently, their fitness might be dependent on the presence of CTL-stimulating viruses in the neighborhood.

Here, we investigate how natural selection determines the evolution of viral innate immunity evasion in the presence of spatial structure. For this, we use a cell automaton in which infection and IFN-mediated immunity occur as diffusion-reaction processes. From these simulations, we infer direct and indirect fitness effects associated to IFN shutdown, as well as quantitative descriptors of spatial structure. Within this framework, we analyze how viral demography, the timing of infection and immunity, and the physical properties of the virions and the medium should determine the evolution of IFN evasion.

### Model

**Fig 1:**
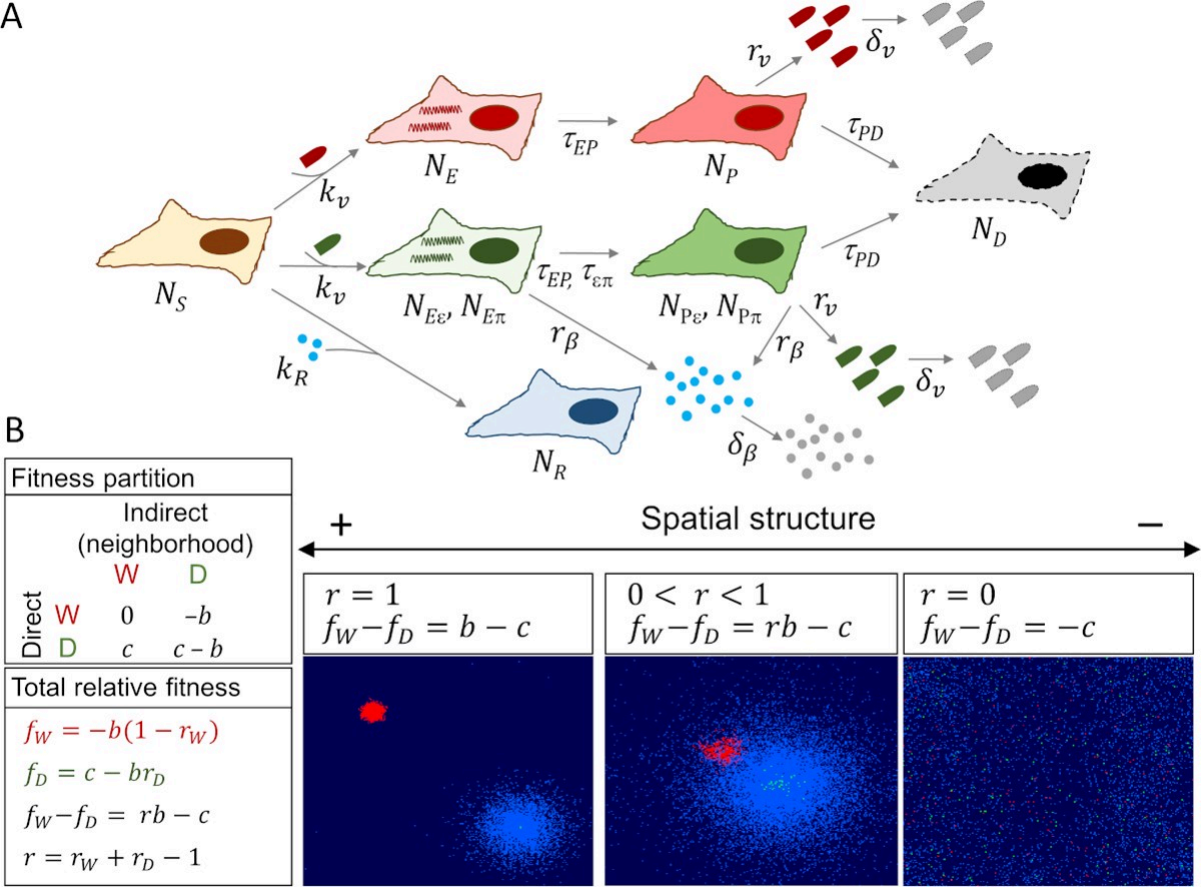
Model. **A**. Scheme of the infection and immunization processes. *N*_*S*_: susceptible (non-infected) cells. *N*_*E*_: infected cells in eclipse phase. *N*_*P*_: virion-producing cells. *N*_*D*_: cells killed by the virus. *N*_*R*_: immunized cells (blue). W-infected cells: red. D-infected cells: green. An eclipse phase between viral sensing (*N*_*ε*_) and IFN secretion (*N*_*π*_) is also considered. The relative speed of infection and IFN production can vary. Hence, D-infected cells can be in four possible stages (*N*_*Eε*_, *N*_*Pε*_, *N*_*Eπ*_, *N*_*Pπ*_). *k*_*v*_: virion infectivity (infection rate). *k*_*R*_: IFN immunization rate. *τ*_*EP*_: viral eclipse half time. *τ*_*επ*_: half time between infection and IFN secretion. *τ*_*PD*_: half time between virion production and cell death. Hence, *τ*_*PD*_ +*τ*_*EP*_ is the total duration of the infection cycle. *r*_*v*_(*W*) and *r*_*v*_(*D*): virion production rates for W and D, respectively. *K* = *r*_*v*_*τ*_*PD*_ is thus the number of virions produced per infected cell. *δ*_*V*_: virion degradation/outflow rate. *r*_*β*_: IFN production rate of immunized cells. *δ*_*β*_: IFN degradation/outflow rate. Values for each parameter are provided in **Table 1**. In this model, post-infection IFN effects are not allowed. **S2 Fig** shows a model with IFN-induced apoptosis. **B.** Fitness partition according to direct and indirect (neighborhood) components. For the W virus in a W neighborhood, *f*_*W*|*W*_ = 0. The term *c* is the direct fitness effect of being an IFN-suppressor (*c* > 0: IFN suppression is costly; *c* < 0: IFN suppression is beneficial). For a D virus in an W neighborhood, *f*_*D*|*W*_ = *c*. The term *b* is the indirect benefit of being in an IFN-suppressive (W) neighborhood, independent of direct effects. The derivation of the overall relative fitness of each variant (*f*_*W*_ and *f*_*D*_) is shown in the text. *r*_*W*_ quantifies how strongly W viruses are influenced by neighbors of the same type, and analogously for *r*_*D*_. The term *r* measures the spatial segregation between W and D in terms of infection and immunity. Three possible scenarios are shown (red: W-infected cells, green: D-infected, light blue: IFN-immunized cells). Left: full segregation (*r* = 1). Center: partial segregation (0 < *r* < 1). Right: complete mixing (*r* = 0).

We considered *N*_*S*_ cells susceptible to infection by a virus (uninfected), *N*_*E*_ infected cells that are not yet virion-producers (eclipse phase), *N*_*P*_ virus-producer cells, and *N*_*D*_ cells killed by the virus (**Fig 1A**). Killed cells were not replaced by new cells and hence became equivalent to empty positions in the grid. We considered two virus variants, W and D. For simplicity, we ignored cells co-infected with both variants. The W variant blocked or did not stimulate IFN production (actively preventing IFN production or avoiding recognition by the innate immune system were equivalent in the context of this model). In contrast, cells infected with the D variant detected the virus and became IFN producers. The model allowed the relative speed of infection and IFN production to vary, such that eclipse-phase cells could be primed for IFN production (*N*_*Eε*_) or be IFN producers (*N*_*Eπ*_), and the same applied to D-producing cells (*N*_*Pε*_ and *N*_*Pπ*_, respectively).

Based on known dynamics [24–26], though, we initially assumed that virion release preceded IFN secretion (*N*_*Eπ*_ ≈ 0). We also incorporated the observation that uninfected cells respond to IFN in a dose-dependent manner [5, 27] and become immunized (*N*_*R*_), but do not produce IFN themselves [28]. Infection (*N*_*S*_→*N*_*E*_) and immunization (*N*_*S*_→*N*_*R*_) were simulated as Poisson stochastic processes occurring with probability

P=1−exp(−λΔt), (1)

for each cell and simulation time unit Δ*t* (0.5 min). For infection, *λ* = *k*_*V*_*VN*_*S*_, where *k*_*V*_ is the infection rate (infectivity), *V* the local virion concentration and *N*_*S*_ equals 1 or 0. For immunization, *λ* = *k*_*R*_*βN*_*S*_, where *k*_*R*_ is the immunization rate, *β* the local IFN concentration, and *N*_*S*_ equals 1 or 0. All other cellular state transitions were modeled as random processes occurring with cumulative probability *P* = 0 for *t* < *τ*, *P* = 0.5 for *t* = *τ*, and *P* = 1 for *t* > *τ*, where *τ* is the half time of the corresponding transition (e.g. *τ*_*EP*_ for *N*_*E*_ → *N*_*P*_; **Fig 1A**).

Viral dynamics obeyed a diffusion-reaction process in an orthogonally divided space, the partial derivative of the virion concentration with time in every grid position being

$$\frac{\partial V}{\partial t}=r_{v}\;N_{P}-\delta_{V}V+\Delta V\times D/\Delta x^{2}.$$ (2)

The first two terms correspond to the reaction part, where *r*_*v*_ is the virion production rate of infected cells. For the D virus, the *N*_*P*_ term was replaced with *N*_*Pε*_ + *N*_*Pπ*_ (that is, all D-producing cells regardless of IFN status). Wherein needed, we used different virion production rates for W and D, *r*_*v*_(*W*) and *r*_*v*_(*D*), respectively. The *δ*_*V*_ parameter is the virion degradation/outflow rate. We ignored the loss of virions due to adsorption to cells. The last term describes diffusion, where *D* is the Stokes-Einstein diffusion coefficient, *Δx* is the grid size and Δ*V* the virion concentration difference between grid subunits. To calculate it, we considered only adjacent cells for each grid position (*x*, *y*), such that:

ΔV=V(x+1,y)+V(x−1,y)+V(x,y−1)+V(x,y+1)−4V(x,y). (3)

In the limits of the grid, we used a continuous-system approach, meaning that the neighbors of the rightmost cells were the leftmost cells, and the neighbors of the uppermost cells were the bottommost cells. For reasons of computational efficiency simulations were performed using a time unit Δ*t* = 0.5 min, but a finer time resolution was needed to calculate the diffusion process. We thus used shorter time units for the diffusion part only and assumed interim quasi-steady states for all other variables in the system.

Analogously, for IFN:

$$\frac{\partial \beta }{\partial t}=r_{\beta }N_{\pi }-\delta_{\beta }\beta +\Delta \beta \times D/\Delta x^{2}.$$ (4)

Viral spread occurred exclusively through the above diffusion-reaction process (for illustration, **S1 Fig** shows the diffusion process of virions and IFN alone, without the reaction term). We did not consider other types of spread, such as cell-to-cell spread, active transport of viruses, or cell mobility. Cellular proliferation was also ignored, since the time scale of the infection was shorter than the typical cell division time of normal (non-tumoral) cells, with some exceptions (e.g activated lymphocytes). For simplicity, we also ignored details of the intracellular infection and immunization processes, and we assumed that, in each cell, after the eclipse phase both IFN and virion production was linear with time. In nature, viruses can follow different replication mechanisms [29] and, hence, virion release is not necessarily linear with time. Also, IFN production is regulated by positive feedback loops in the infected cell [30], which we did not consider.

Initially, we assumed that IFN had no effect on already infected cells but we later relaxed this assumption to include autocrine effects as well as post-infection paracrine effects. For this, we allowed infected cells to respond to IFN by undergoing apoptosis (**S2 Fig**; *N*_*A*_ cells). We modeled IFN-triggered cell death (*N*_*E*_→*N*_*A*_, *N*_*P*_→*N*_*A*_) as a Poisson stochastic process as above, with *λ* = *k*_*A*_*βN*_*P*_ for virion-producing cells and *λ* = *k*_*A*_*βN*_*E*_ for cells in eclipse phase, where *k*_*A*_ is the IFN-induced apoptosis rate of infected cells.

The cellular automaton diffusion-reaction simulations were performed using MATLAB R2018b scripts (**S1 File**).

We calculated the growth rate of each virus variant as

$$R(t)=\log\left(\frac{N_{I}}{N_{I(0)}}\right)/t,$$ (5)

where *N*_*I*(0)_ is the initial number of infected cells and *N*_*I*_ the number of cells infected at time *t* including both producer and eclipse-phase cells (*N*_*I*_ = *N*_*E*_ + *N*_*P*_ for the W variant; *N*_*I*_ = *N*_*Eε*_ + *N*_*Eπ*_ + *N*_*Pε*_ + *N*_*Pπ*_ for the D variant). We used log_10_ instead of the natural logarithm to facilitate visualization of the results.

To obtain a time-integrated metric of viral fitness, we calculated $R^{*}=\log\left(\frac{N^{*}}{N_{I(0)}}\right)/t_{f}$, where N* is the cumulative number of cells infected throughout the simulation and *t*_*f*_ the final time point (36 h).

Following our previous work [5], we defined the fitness (*f*) of a focal virus conditional to the presence of other viruses in the same population (neighborhood; **Fig 1B**). For this, we used the pure W infection (i.e. W virus in a W neighborhood) as reference and we expressed fitness relative to it, such that:

$$f=R-R_{W|W},$$ (6)

where *R*_*W*|*W*_ is the growth rate of a pure W infection, or

$$f=R^{*}-R_{W|W}^{*},$$ (7)

for time-integrated fitness. Therefore, by definition pure W infections had fitness *f* = 0. Let us then consider the extreme situation in which, due to the presence of D neighbors, the spread of the W virus would be hampered by as many IFN-immunized cells as in a pure D infection. For this scenario, we defined the fitness of W as *f*_*W*|*D*_ = −*b* (W virus in D neighborhood). Hence, −*b* is the cost of being in a D neighborhood and, reversely, *b* is the benefit of being in a W neighborhood. Although *b* depends on paracrine signaling (that is, the benefit of avoiding IFN secretion), it may also be indirectly influenced by other features of the neighborhood such as, for instance, local virion abundance, which determines competition for cellular resources. Next, let us consider a situation in which the spread of a D virus would not be hampered by the immunization of neighbor cells (D virus in W neighborhood). We defined the fitness of D infections accordingly as *f*_*D*|*W*_ = *c*. Here, *c* can be interpreted as the direct effect of blocking IFN for the focal virus, independent of neighborhood. If *c* > 0, blocking IFN has a direct cost, whereas if *c* < 0 blocking IFN has a direct benefit. Finally, we defined the fitness of a D virus in a D neighborhood (pure D infection) as *f*_*D*|*D*_ = *c*−*b*, which is simply the sum of the independent direct and indirect terms.

Having considered the four extreme cases in which the neighborhood was purely D or purely W, we then allowed for intermediate scenarios (**Fig 1B**). For this, we defined the overall fitness of W as

$$f_{W}=r_{W}f_{W|W}+(1-r_{W})f_{W|D},$$ (8)

where *r*_*W*_ is a parameter that quantifies how strongly W viruses are influenced by W neighbors. It follows that *f*_*W*_ = −*b*(1−*r*_*W*_). Analogously, we defined *f*_*D*_ = *r*_*D*_*f*_*D*|*D*_+(1−*r*_*D*_)*f*_*D*|*W*_, where *r*_*D*_ quantifies how strongly D viruses are influenced by D neighbors. Thus, *f*_*D*_ = *c*−*r*_*D*_*b*. Natural selection will favor the W variant and, therefore, will promote the evolution of IFN evasion if *f*_*W*_−*f*_*D*_>0. Consequently:

$$f_{W}-f_{D}=b(r_{W}+r_{D}-1)-c.$$ (9)

The quantity *r*_*W*_+*r*_*D*_−1 describes the difference between the neighborhoods of W and D. More precisely, *r*_*W*_+*r*_*D*_−1 is the difference between the indirect IFN-mediated effects experienced by W and D viruses. By denoting *r* = *r*_*W*_+*r*_*D*_−1, the condition for the evolution of IFN shutdown is:

rb−c>0. (10)

This expression corresponds to Hamilton´s rule [31]. Hence, the condition for natural selection to favor viruses that block or avoid IFN signaling can be written in terms of classical social evolution theory. In the social evolution field, *r* has been often defined as the genetic relatedness between interacting partners for the relevant trait. In our context, such relatedness depends strictly on the spatial structure of infection and immunity. If the two variants are fully insulated, the benefits/costs of preventing/stimulating paracrine signaling will be felt only by viruses of the same kind as the focal virus and, hence, *r* = 1 (**Fig 1B**). In contrast, if the innate immune response triggered by the IFN-stimulating variant affects both variants equally, *r* = 0. If IFN-blocking and IFN-stimulating viruses are spatially segregated to some extent (*r* > 0), selection may indirectly favor IFN-blocking viruses because they take a greater share of the benefits of preventing paracrine signaling. Notice that all parameters considered (*b*, *c*, *r*_*W*_, *r*_*D*_, and *r*) are time-dependent because fitness and spatial structure vary as the infection and the immune response progress. Also, these parameters are defined at the population level, and are not inferred from an analysis of each individual cell in the grid but, instead, from total cell counts in a given population. An Excel spreadsheet for the calculation of these parameters is available upon request.

## Results

### Selection for IFN shutdown is determined by spatial structure

We investigated the dynamics of viral spread and innate immune responses in a square grid containing 47,961 cells. Our model considered virion and IFN diffusion as well as cell infection and immunization (**Fig 1A**). For this, we used the empirically-determined size and degradation rate of VSV particles and the measured diffusion coefficient of chicken IFN. Generally, parameter values corresponded to a rapidly growing lytic virus and adherent IFN-producing cells. Yet, the model is generally applicable to different types of viruses. Details of parameter values are provided in **Table 1**.

**Table 1:** Default parameters used in the simulations.

Process	Parameter	Value	Units	Definition
Diffusion	*A*	1.2	cm^2^	Area of the cell population
	*N*	4.8 × 10^4^	-	Number of cells (A×C)
	*kT*	4.28 × 10^−21^	J	Boltzmann constant × temperature (37°C)
	*μ*	6.91 × 10^−4^	Pa.s	Dynamic viscosity of the medium (water)
	*D*_*v*_	0.18	*μ*m	Hydrodynamic diameter of the virion^1^
	*D*_*β*_	0.007	*μ*m	Hydrodynamic diameter of IFN^2^
Reaction (infection)	*k*_*v*_	10^−8^	cell^–1^virion^–1^ cm^2^ min^–1^	Virion infectivity^3^
	*δ*_*v*_	0.002	min^–1^	Virion degradation/outflow rate^4^
	*τ*_*EP*_	6	h	Eclipse phase half time^5^
	*τ*_*PD*_	6	h	Virion production half time^6^
	*r*_*v*_	0.28	virions cell^–1^ min^–1^	Virion production rate^7^
Reaction (immunity)	*τ*_*επ*_	9	h	Virus entry to IFN production half time^8^
	*r*_*β*_	1	units cm^–2^cell^–1^ min^–1^	Production rate of IFN^9^
	*k*_*R*_	0.01	cell^–1^ min^–1^ unit^–1^ cm^2^	Immunization rate^10^
	*δ*_*β*_	2 × 10^−4^	min^–1^	IFN degradation/outflow rate^11^

^1^ Size of a VSV particle [32].

^2^ Inferred from the dynamic diffusion constant of chicken IFN using the Stokes-Einstein equation [33].

^3^ Approximately 1/24 virions successfully infect cells after 1 h, i.e. a particle-to-foci ratio of 24 in a typical infectivity assay. This ratio varies amply among viruses, from close to 1 to 1:1000 [34].

^4^A half time of approximately 18 h, as estimated for VSV [35].

^5^A typical eclipse time for many rapidly replicating animal viruses.

^6^Total duration of the infection cycle set to τ_EP_ + τ_PD_ = 12 h, a typical value for many animal viruses.

^7^Adjusted to produce *r*_*v*_*τ*_*PD*_ = 100 virions/cell in two dimensions. In three dimensions, this would scale up to 100^3/2^ = 1000 virions per cell, a typical value for an animal virus.

^8^Based on the observation that IFN starts to be released after virions [24, 25] and on IFN production kinetics [26].

^9^In arbitrary units; an entire cell population infected with the D virus would produce approximately 200 units (or 200^3/2^ = 2800 units in three dimensions).

^10^Such that 1 unit immunizes ca. 50% of the cells in 1h.

^11^Empirically determined stability of IFN [26].

**Fig 2:**
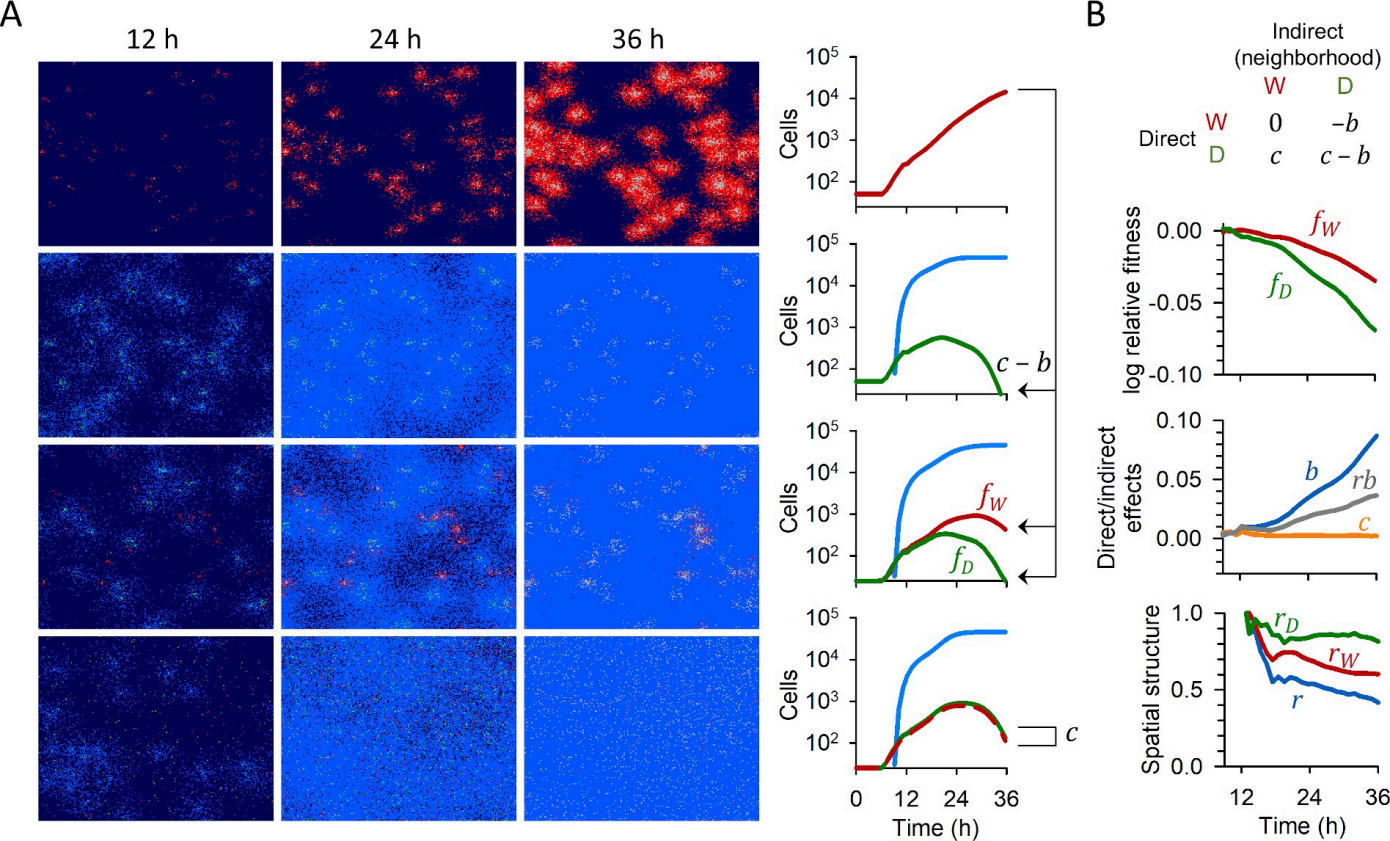
Dynamics of the infection-immunization process and inference of fitness components. **A.** Left: spatial structure of the infection and immune response at different time points. Right: cell counts. Susceptible cells are shown in dark blue (cell counts not shown), cells infected with the W virus in red, cells infected with the D virus in green, immunized cells in light blue, and dead cells in white (counts now shown). Infections were initiated with 50 infected cells of pure W, pure D, or a 1:1 mix of W and D. The last row shows an unstructured infection (*r* = 0) in which the concentration of virions was homogenized at every time unit of the simulation. Parameter values are given in **Table 1**. **B.** Inferred fitness components. Top graph: fitness values of W and D in mixed infections relative to pure W infections (*f*_*W*_ and *f*_*D*_). Middle: inferred direct (*c*) and indirect (*b*) fitness components. For pure D infections *f*_*D*_ = *f*_*D*|*D*_ = *c*−*b* whereas, in mixed infections lacking spatial structure, *f*_*W*_−*f*_*D*_ = *-c*. This allows *c* and *b* to be inferred at each time point. In mixed infections exhibiting spatial structure *f*_*W*_−*f*_*D*_ = *rb*−*c*, which gives the *rb* term. Bottom: parameters describing spatial structure. These were calculated as *r*_*W*_ = 1+*f*_*W*_/*b*, *r*_*D*_ = (*c*−*f*_*D*_)/*b*, and *r* = *r*_*W*_+*r*_*D*_−1. The plots of all parameters are averages obtained from 10 simulations. The time range shown encompasses from the start of IFN release (9 h) to the approximate time point in which the pure D infection died out (36 h, endpoint). The spatial structure parameters could not be measured reliably before 13 h because *b* was too close to 0. In these simulations, IFNs suppression had no direct fitness effects (*c* = 0).

The simulated infections progressed as foci as a result of the diffusion-reaction process, reproducing the typical spread mode of many viruses (**Fig 2A**). In simulations containing only one type of virus variant, IFN-blocking (W) and IFN-stimulating (D) viruses initially spread at similar rates. However, D infections subsequently became halted by innate immunity, whereas W infections progressed and invaded the entire cell population. In mixed infections initiated with an equal input of W and D, the growth of both variants was halted as the immune response was deployed (**Fig 2A**). Hence, in mixed infections the fitness of W and D relative to a pure W infection (*f*_*W*_ and *f*_*D*_, respectively) decreased with time.

The fitness difference between W and D in mixed infections can be expressed as *f*_*W*_−*f*_*D*_ = *rb*−*c* (see Model; **Fig 1B**) where *b* is the indirect benefit of being in a W, IFN suppressive neighborhood, *c* is the direct fitness effect of suppressing IFN on the focal virus, and *r* is the difference between the neighborhoods of W and D determined by spatial structure. In the absence of spatial structure, *r* = 0 and hence *f*_*W*_−*f*_*D*_ = *-c*. Hence, to infer *c* we performed simulations of mixed infections in which spatial structure was disrupted by equalizing virion concentration in the grid at every time unit Δ*t* (0.5 min). Knowing *c*, we obtained *b* by comparing the fitness of a pure D infection relative to a pure W infection, *f*_*D*|*D*_ = *c*−*b* (**Fig 2B**). In these simulations, IFN had no effect on already infected cells and, hence, autocrine signaling was not allowed. Consequently, we obtained *c* = 0. In contrast, *b* increased with time as the cell population became immunized.

In mixed infections, initially *r* was close to 1 because each virus was only weakly influenced by neighbors from other infection foci. Yet, as the infection progressed and IFN-mediated immunity expanded throughout the cell population, *r*-values decreased (**Fig 2B**). We calculated the parameters describing spatial structure associated to each variant as *r*_*W*_ = 1+*f*_*W*_/*b*, *r*_*D*_ = (*c*−*f*_*D*_)/*b*, and *r* = *r*_*W*_+*r*_*D*_−1, where *r*_*W*_ describes to what extent the fitness of W viruses is determined by neighbor viruses of the same type W and, analogously, *r*_*D*_ describes to what extent the fitness of D viruses is determined by neighbor viruses of the same type D (see Model for details; **Fig 1B**). We found that *r*_*W*_ decayed with time faster than *r*_*D*_, indicating that the neighborhood of both variants gradually became more similar to a pure D infection than to a pure W infection. This shows that IFN functions as a harmful diffusible molecule for the virus, akin to a pollutant or poison. Thus, we define IFN as a “public bad”, by contraposition to well-known diffusible public goods (e.g. microbial secreted enzymes).

We ended simulations at 36 h, a time point at which pure D infections died out, and calculated a time-integrated fitness value using the cumulative number of infected cells (N*), from which we obtained the corresponding time-integrated values of each parameter in our model (**Table 2**). Despite the inhibitory effects of IFN, W remained fitter than D. Because *c* = 0, this was strictly due to spatial structure which, in turn, emerged from the diffusion-reaction infection and immunity processes and allowed W to be on average less adversely affected by IFN-mediated immunity than D. These results suggest that spatial structure is a key determinant of the evolution of IFN suppression in viruses.

**Table 2:** Fitness and spatial structure descriptors inferred from 10 replicate simulations using parameter values provided in Table 1.

	Parameter	Mean ± SEM
Pure infections	$N_{\left(W|W\right)}^{*}$	18,108 **±** 141 cells
	$N_{\left(D|D\right)}^{*}$	677 **±** 17 cells
Mixed infections	$N_{\left(W\right)}^{*}$	1187 **±** 66 cells
	$N_{\left(D\right)}^{*}$	478 **±** 14 cells
Mixed infections “shaked” (*r* = 0)	$N_{\left(W\right)}^{*}$	1146 **±** 29 cells
	$N_{\left(D\right)}^{*}$	1132 **±** 16 cells
Inferred fitness and spatial structure descriptors	*f*_*W*_	– 0.0247 **±** 0.0006
	*f*_*D*_	– 0.0355 **±** 0.0004
	*b*	0.395 **±** 0.0006
	*c*	– 0.0001 **±** 0.0003
	*rb*	0.0107 **±** 0.0009
	*r*_*W*_	0.3753 **±** 0.0173
	*r*_*D*_	0.8957 **±** 0.0083
	*r*	0.2710 **±** 0.0211

### Factors promoting spatial insulation of IFN-suppressing and IFN-stimulating variants

**Fig 3:**
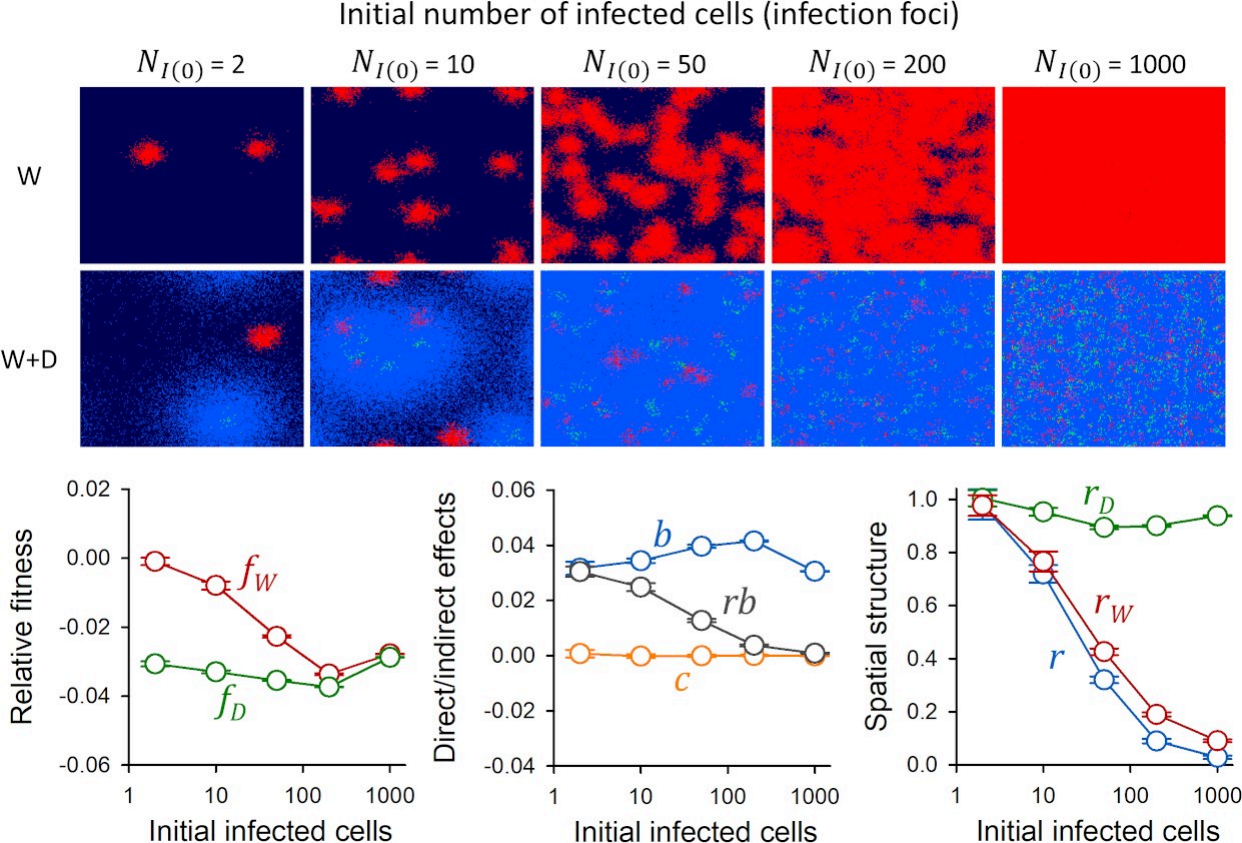
Effect of the founder population size on the relative fitness of IFN supressing and IFN-stimulating virus variants. Top: spatial structure of the infection at endpoint (36 h) for different numbers of initially infected cells, ranging from 2 to 1000. Pure W infections and mixed (W+D) infections are shown. Susceptible cells are shown in dark blue, cells infected with the W virus in red, cells infected with the D virus in green, and immunized cells in light blue. Infected cells are shown in a cumulative manner, meaning that all cells infected with each variant throughout the progression of the infection are depicted. Thus, dead cells are not shown. Bottom: time-integrated fitness components calculated using the cumulative number of cells infected with each variant in mixed infections, shown as a function of the initial number of infected cells (mean ± SEM values from ten replicate simulations are shown). Left: fitness of W and D relative to pure W infections. Center: direct and indirect fitness components. Right: descriptors of spatial structure.

As shown above, the fitness of IFN-suppressing virus variants depends on their spatial insulation from IFN-stimulating variants. A straightforward factor determining such segregation is the initial fraction of infected cells. To illustrate this, we performed simulations in which we varied the initial number of infected cells from *N*_*I*(0)_ = 2 to *N*_*I*(0)_ = 1000, keeping constant the initial frequency of each variant at 50% as well as the initial number of susceptible cells. We found that for *N*_*I*(0)_ = 2, the W variant was largely unaffected by the presence of the D virus, whereas for *N*_*I*(0)_ = 1000 the interference was such that both variants showed similarly low fitness (**Fig 3**). The relative fitness of the D variant also decayed as *N*_*I*(0)_ increased due to self-interference (except for *N*_*I*(0)_ = 1000, due to saturation of W spread). The indirect benefit of suppressing IFN secretion (*b*) tended to increase with *N*_*I*(0)_ because larger *N*_*I*(0)_ values produced more immunized cells in the absence of IFN suppression. However, spatial structure dropped from *r* ≈ 1 for *N*_*I*(0)_ = 2 to *r* ≈ 0 for *N*_*I*(0)_ = 1000. This effect was driven by a gradual populating of the W neighborhood with IFN-immunized cells, as shown by the decreasing *r*_*W*_ values. Spatial structure determined the fitness advantage of W over D through the *rb* term, such that selection for IFN suppression was stronger for lower *N*_*I*(0)_ values. These results suggest that IFN evasion should more easily evolve in viruses that experience severe population bottlenecks during transmission and hence produce few, isolated initial infection foci.

Another factor that should determine selection for IFN suppression is medium viscosity, since viscosity determines the diffusion coefficient of IFN and virions. We found that the spatial structure parameter *r* increased with medium viscosity. The more local nature of infection and immunization at higher viscosities allowed the W variant to be less adversely affected by D viruses, increasing the fitness difference between W and D and thus making selection for IFN suppression stronger (**S3 Fig**). We also set out to test whether selection for IFN suppression depended on virion size. For this, we performed simulations in which we varied virion size from 50 nm to 500 nm. We found that the spatial structure parameter *r* tended to increase with larger virion sizes, but that this effect was weak (**S4 Fig**) because IFN diffusion was unaffected.

### IFN suppression as an altruistic trait

**Fig 4:**
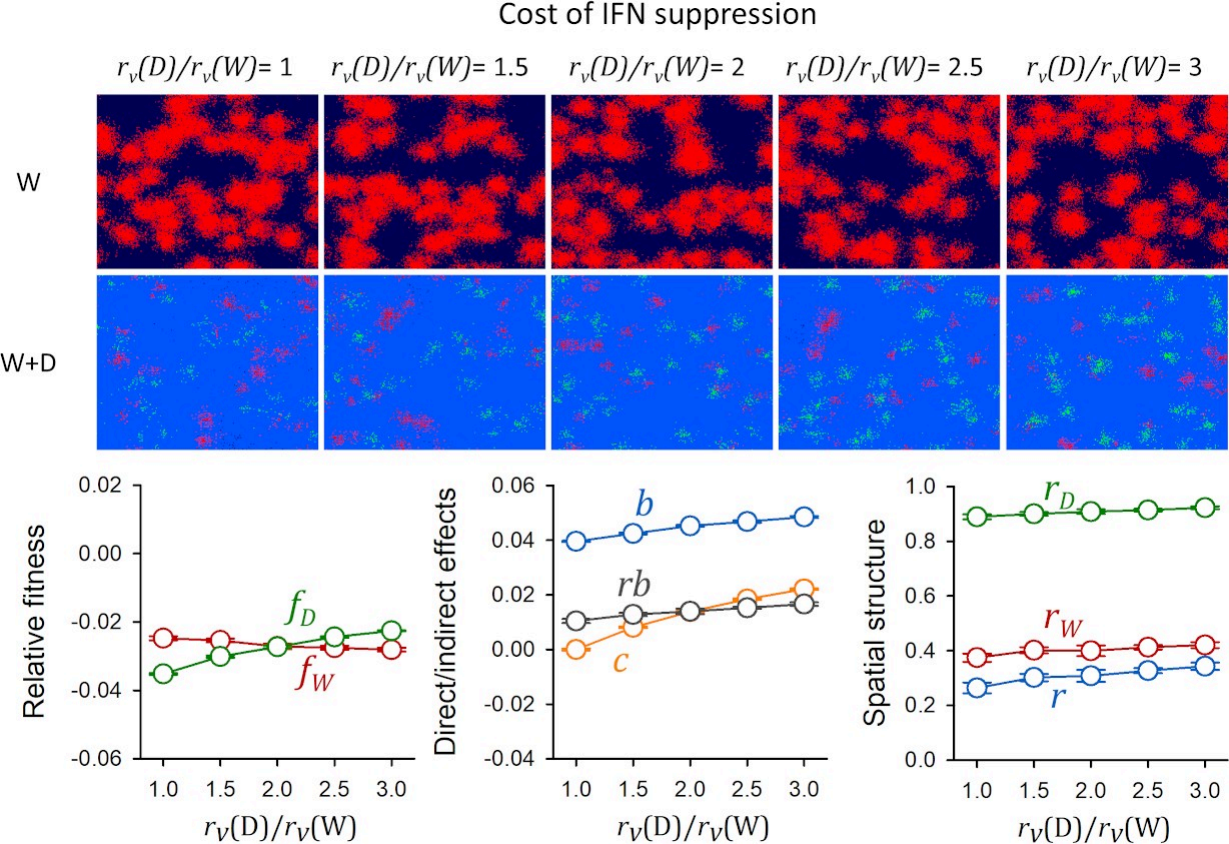
Indirect selection of costly IFN suppression (altruism). Top: spatial structure of the infection at endpoint (36 h) as a function of the cost associated to IFN suppression. This cost was implemented as an excess virion production rate for IFN-stimulating viruses compared to IFN-suppressing viruses. Pure W infections and mixed (W+D) infections are shown. Susceptible cells are shown in dark blue, cells infected with the W virus in red, cells infected with the D virus in green, and immunized cells in light blue. Infected cells are shown in a cumulative manner, meaning that all cells infected with each variant throughout the progression of the infection are depicted. Thus, dead cells are not shown. Bottom: time-integrated fitness components calculated using the cumulative number of cells infected with each variant in mixed infections, shown as a function of the cost of IFN suppression (mean ± SEM values from ten replicate simulations). Left: fitness of W and D relative to pure W infections. Center: direct and indirect fitness components. Right: descriptors of spatial structure.

The above results indicate that IFN shutdown can be favored by natural selection acting on indirect fitness effects, as long as infection and immunity are spatially structured. This suggests that IFN shutdown could evolve even if it imposes a direct fitness cost to the actor (*c* > 0). From a social evolution perspective, this implies that innate immunity evasion could evolve as an altruistic trait. To illustrate this, we made IFN shutdown costly by assigning a direct advantage to D in terms of virion production rate (higher *r*_*v*_ and thus, higher number of virions produced per cell). Initially, D expanded faster than W throughout the cell population. However, as cells became immunized, the spread of D was halted whereas W continued to expand (**S5 Fig**). Using the cumulative number of infected cells to calculate fitness as above, we found that W remained fitter than D for a virion production cost of up to twofold, i.e. *r*_*v*_(*D*) = 2*r*_*v*_(*W*) (**Fig 4**). As expected, *c*-values increased as the advantage of D in terms of virion productivity became larger. Less intuitively, *b* also increased despite the fact that parameters controlling paracrine innate immunity were not changed. This occurred because D neighborhoods contained more virions than W neighborhoods and hence experienced stronger local competition for cells. Hence, *b* captured fitness effects associated to spatial structure including but not limited to paracrine signaling. To show this, we repeated the above simulations switching innate immunity off (*k*_*R*_ = 0). Under these conditions, D outcompeted W owing to its higher virion production rate. Yet, again, *b* increased as the excess virion production of D became larger, even if no paracrine response was possible, confirming that *b* captured the effect of local competition for cells (**S6 Fig**).

### Contribution of autocrine signaling to direct fitness

To explore the situation in which IFN blockade provides a direct benefit (*c* < 0), we allowed for autocrine IFN signaling. To implement this, infected cells responded to IFN by undergoing apoptosis (**S2 Fig**). This also allowed infected cells to respond to IFN secreted by other cells (post-infection paracrine signaling). Without changing the rate at which IFN made non-infected cells immune (*k*_*R*_), we increased the rate at which IFN triggered apoptosis (*k*_*A*_). This showed that, even for relatively strong post-infection IFN effects, autocrine signaling had a minimal impact on the direct fitness component *c*, which was always close to zero and much smaller than the indirect components *b* or *rb* (**S7 Fig**). The reason for this is that virion production preceded IFN responses, such that IFN signaling on already infected cells was relatively unimportant. This is in line with our recent experimental results, which indicated a low efficacy of IFN (autocrine or paracrine) in already infected cells [5].

**Fig 5:**
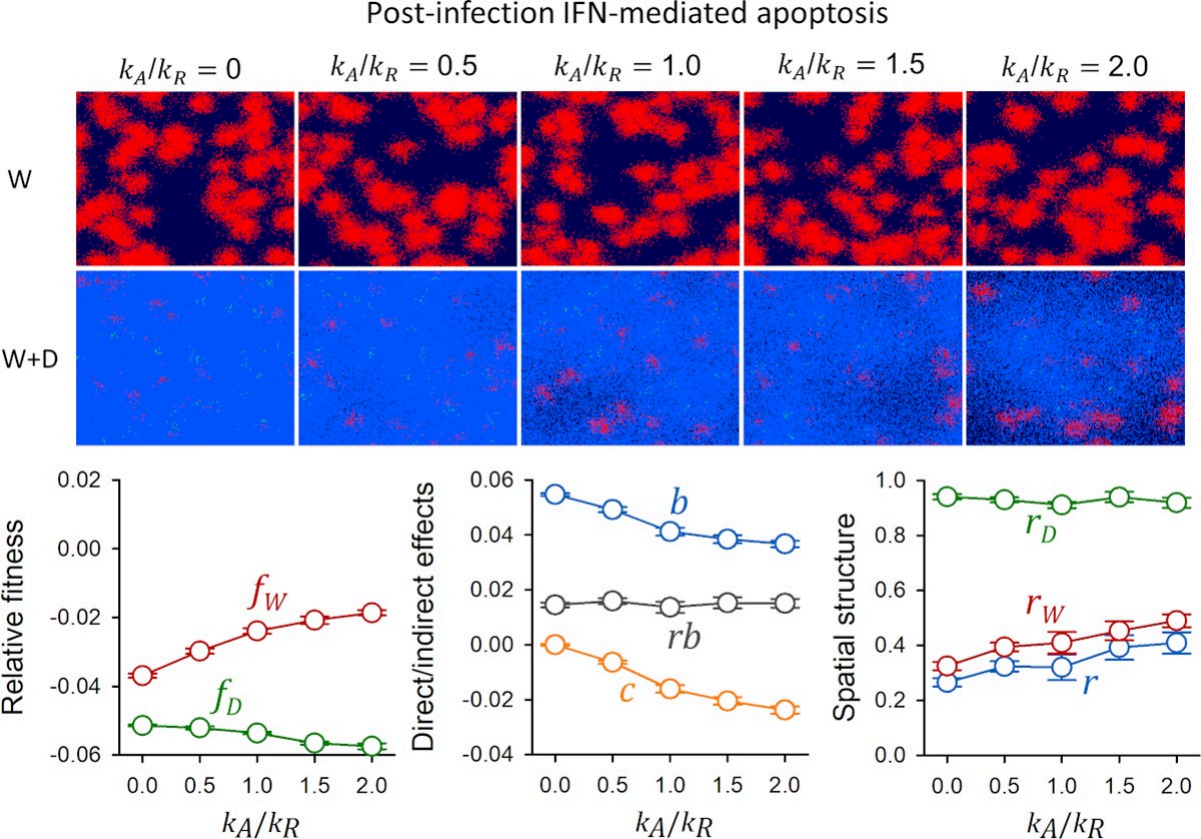
Effect of IFN-mediated post-infection apoptosis on the fitness of IFN supressing and IFN-stimulating virus variants assuming that IFN secretion precedes virion release. Top: spatial structure of the infection at endpoint (36 h) as a function of the rate of IFN-mediated apoptosis (*k*_*A*_) relative to IFN-mediated immunization (*k*_*R*_). Pure W infections and mixed (W+D) infections are shown. Susceptible cells are shown in dark blue, cells infected with the W virus in red, cells infected with the D virus in green, and immunized cells in light blue. Infected cells are shown in a cumulative manner, meaning that all cells infected with each variant throughout the progression of the infection are depicted. Thus, dead cells are not shown. Bottom: time-integrated fitness components calculated using the cumulative number of cells infected with each variant in mixed infections, shown as the ratio between the rate of IFN-mediated apoptosis and the rate of IFN-mediated immunization *k*_*A*_/*k*_*R*_ (mean ± SEM values from ten replicate simulations). Left: fitness of W and D relative to pure W infections. Center: direct and indirect fitness components. Right: descriptors of spatial structure.

In contrast, when we allowed IFN to be released before virions (5 h versus 6 h) and IFN-triggered apoptosis was strong (*k*_*A*_>*k*_*R*_), *b* decreased and–*c* increased by the same amount (**Fig 5**). Hence, the indirect benefits of blocking IFN secretion partially became direct benefits. Despite this drop in *b*, the *rb* fitness component was largely insensitive to changes in *k*_*A*_. This occurred because, as *k*_*A*_ increased, D infections became more localized and thus produced fewer immunized cells. This increased spatial structure and allowed the W virus to be less severely affected by the presence of D, as shown by the greater *r*_*W*_ and *r* values. As a result, the *rb* and–*c* terms were similarly large, indicating that spatial structure-dependent indirect fitness effects contributed significantly to the benefits of IFN suppression even in the presence of strong post-infection IFN-induced apoptosis.

## Discussion

In previous work, we showed that IFN shutdown can be considered a social process because the IFN-stimulating status of a given virus modifies the fitness of neighbor viruses [5]. Specifically, mutants that stimulated IFN production had a negative impact on the fitness of IFN-stimulating and IFN-suppressing members of the viral population, whereas IFN blockers provided little or no benefits to IFN stimulators. Hence, from the virus perspective, IFN functions as a diffusible “public bad”, and the ability of natural selection to promote IFN shutdown depends critically on spatial structure. Here, we have investigated this process quantitatively using a biophysically well-defined system in which a diffusion-reaction model was implemented in cell automaton simulations. We have shown that spatial structure in the form of infection and immunization foci can emerge from IFN and virion concentration gradients, which in turn depend on demography (e.g. transmission bottlenecks) and the properties of the medium (e.g. viscosity). Because IFN is smaller and hence diffuses faster than virions, immunization can reach larger areas than infection, even if cells release virions faster than IFN. Delayed but effective innate immune responses capable of successfully arresting virus progression by producing a ring of immunized cells around infection foci have been experimentally shown previously using IFN-stimulating virus variants [24].

We found that, whenever virion release precedes IFN secretion, the fitness associated to IFN evasion should not be strongly determined by direct effects exerted on the infected cell, but rather by indirect (neighborhood) effects. An important consequence of indirect fitness effects being larger than direct effects is that IFN evasion could be favored by natural selection even if costly for the actor, meaning that IFN evasion could evolve as an altruistic trait. Cooperation and altruism have long been studied by evolutionary biologists [31, 36–40]. In principle, natural selection should not favor traits that diminish individual fitness because cooperators can be invaded by cheaters that benefit from cooperative traits without reciprocating. However, cooperation breakdown can be avoided if cooperators tend to interact preferentially with other cooperators. Spatial structure allows for such assortment in a wide variety of organisms and is hence considered a fundamental factor driving the evolution of cooperation [41–44]. Spatial structure should actually be the main driver of cooperation in simple organisms for which other mechanisms of assortment such as learning and memory are not possible, as is the case of viruses. Here, we have shown that particle diffusion provides a simple and general physical basis for cooperator assortment in viruses. Within the context of innate immunity evasion, in mixed populations containing both IFN-stimulating and IFN-suppressing virus variants, IFN and virion concentration gradients allow IFN suppressors to be less severely affected by immunization than IFN stimulators.

The role of spatial structure in the evolution of innate immunity evasion should be particularly critical when shutting down IFN entails a direct fitness cost, since IFN suppression then becomes an altruistic trait. In future work, it would be interesting to elucidate the direct and indirect fitness effects of IFN suppression in different viruses, and the mechanisms involved in these effects. Yet, we found that in the absence of such direct costs, and even when IFN suppression has direct benefits, the evolution of IFN shutdown should depend strongly on spatial structure, provided that the ability of IFN to halt viral spread is mainly determined by paracrine signaling. As we have shown, the importance of spatial structure increases if virion release precedes IFN signaling. Currently available empirical evidence supports the view that this is the case for different viruses [24, 25]. Although further work is required to establish more general conclusions, virion release prior to IFN signaling should be frequent in viral infections because rapid replication and progeny release tend to be critical for the survival of many viruses.

Spatial structure is pervasive in viral infections. In addition to the basic diffusion-reaction process investigated here, spatial structure can result from other processes such as localized cell-to-cell virus transfer, subversion of cellular structures for enhancing viral spread, use of mobile cells for disseminating the infection, limited virus trafficking among organs or body compartments, inter-host transmission bottlenecks, and so on [45–50]. In future work it would be interesting to investigate the validity of our results in these more complex frameworks. Other complex scenarios, such as tridimensional structures and the spread of the virus and cytokines through vessels could be considered. A common qualitative observation, though, is that viral infections often exhibit metapopulation structures in which quasi-isolated demes are initiated by small number of founder viral particles, increasing genetic drift and intra-deme genetic relatedness. This should make the evolution of cooperative traits such as IFN evasion more likely. In some cases, though, spatial structure might be less evident, such as in blood-borne viruses at the intra-host level. Potentially, selection for IFN evasion might be less efficient in these viruses, or these viruses may utilize different IFN evasion strategies, such as antagonizing downstream antiviral responses triggered by IFN signaling in infected cells.

## Supporting information

S1 Fig Diffusion model. **A.** Diffusion of particles with a hydrodynamic diameter of 180 nm (virions, left) and 7 nm (IFN, right). **B.** Mean particle concentration with time (in arbitrary units). **C.** Variance of the particle concentration with time (green: 180 nm; blue: 7 nm; dashed lines: intermediate sizes).
(TIF)

S2 Fig Scheme of the infection and immunization processes in a model allowing for post-infection IFN effects. *N*_*S*_: susceptible (non-infected) cells. *N*_*E*_: infected cells in eclipse phase. *N*_*P*_: virion-producing cells. *N*_*D*_: cells killed by the virus. *N*_*R*_: immunized cells (blue). W-infected cells: red. D-infected cells: green. An eclipse phase between viral sensing (*N*_*ε*_) and IFN secretion (*N*_*π*_) is also considered. The relative speed of infection and IFN production can vary. Hence, D-infected cells can be in four possible stages (*N*_*Eε*_, *N*_*Pε*_, *N*_*Eπ*_, *N*_*Pπ*_). *k*_*v*_: virion infectivity (infection rate). *k*_*R*_: IFN immunization rate. *τ*_*EP*_: viral eclipse half time. *τ*_*επ*_: half time between infection and IFN secretion. *τ*_*PD*_: half time between virion production and cell death. Thus *τ*_*PD*_ + *τ*_*EP*_, is the total duration of the infection cycle. *r*_*v*_: virion production rate. *K* = *r*_*v*_*τ*_*PD*_ is thus the number of virions produced per infected cell. *δ*_*V*_: virion degradation/outflow rate. *r*_*β*_: IFN production rate of immunized cells. *δ*_*β*_: IFN degradation/outflow rate. Infected cells also respond to IFN by undergoing apoptosis (*N*_*A*_ cells). *k*_*A*_: IFN-induced apoptosis rate of infected cells.
(TIF)

S3 Fig Effect of medium viscosity on the fitness of IFN supressing and IFN-stimulating virus variants. Top: spatial structure of the infection at endpoint (36 h) for increasing viscosities. Pure W infections and mixed (W+D) infections are shown. Susceptible cells are shown in dark blue, cells infected with the W virus in red, cells infected with the D virus in green, and immunized cells in light blue. Infected cells are shown in a cumulative manner, meaning that all cells infected with each variant throughout the progression of the infection are depicted. Thus, dead cells are not shown. Bottom: time-integrated fitness components calculated using the cumulative number of cells infected with each variant in mixed infections, shown as a function of medium viscosity (mean ± SEM values from ten replicate simulations are shown). Left: fitness of W and D relative to pure W infections. Center: direct and indirect fitness components. Right: descriptors of spatial structure.
(TIF)

S4 Fig Effect of virion size on the fitness of IFN supressing and IFN-stimulating virus variants. Top: spatial structure of the infection at endpoint (36 h) for increasing virion sizes. Pure W infections and mixed (W+D) infections are shown. Susceptible cells are shown in dark blue, cells infected with the W virus in red, cells infected with the D virus in green, and immunized cells in light blue. Infected cells are shown in a cumulative manner, meaning that all cells infected with each variant throughout the progression of the infection are depicted. Thus, dead cells are not shown. Bottom: time-integrated fitness components calculated using the cumulative number of cells infected with each variant in mixed infections, shown as a function of virion size (mean ± SEM values from ten replicate simulations are shown). Left: fitness of W and D relative to pure W infections. Center: direct and indirect fitness components. Right: descriptors of spatial structure.
(TIF)

S5 Fig Dynamics of the infection-immunization process and social evolution parameters with a cost to IFN blockade (*c* > 0). **a.** Spatial structure of the infection and immune response at different time points (left) and cell counts (right). The color legend is as in **Fig 2**. Parameter values are as in **Table 1**, except that virus D produces twice as much progeny virions, i.e. *r*_*v*_(*D*) = 0.56 virions cell^–1^ min^–1^ and *r*_*v*_(*W*) = 0.28 virions cell^–1^ min^–1^. **b.** Inferred fitness components. The twofold excess progeny produced by D results in a direct fitness advantage *c* = 0.026 ± 0.001 that counterbalances the indirect fitness advantage obtained by W (see also **Fig 4**).
(TIF)

S6 Fig Effect of local competition on indirect fitness. In these simulations, the D virus had a fitness advantage over the W virus in terms of an increased virion production rate, but innate immunity was disabled.Top: spatial structure of the infection at endpoint (36 h) as a function of the cost imposed to the W virus. Only mixed (W+D) infections are shown. Susceptible cells are shown in dark blue, cells infected with the W virus in red, and cells infected with the D virus in green. Infected cells are shown in a cumulative manner, meaning that all cells infected with each variant throughout the progression of the infection are depicted. Thus, dead cells are not shown. Bottom: time-integrated fitness components calculated using the cumulative number of cells infected with each variant in mixed infections, shown as a function of the cost imposed to W (mean ± SEM values from ten replicate simulations). Left: fitness of W and D relative to pure W infections. Center: direct and indirect fitness components. Right: descriptors of spatial structure.
(TIF)

S7 Fig Effect of the IFN-mediated post-infection apoptosis on the fitness of IFN supressing and IFN-stimulating virus variants assuming that virion release precedes IFN secretion. Top: spatial structure of the infection at endpoint (36 h) as a function of the rate of IFN-mediated apoptosis (*k*_*A*_) relative to IFN-mediated immunization (*k*_*R*_). Pure W infections and mixed (W+D) infections are shown. Susceptible cells are shown in dark blue, cells infected with the W virus in red, cells infected with the D virus in green, and immunized cells in light blue. Infected cells are shown in a cumulative manner, meaning that all cells infected with each variant throughout the progression of the infection are depicted. Thus, dead cells are not shown. Bottom: time-integrated fitness components calculated using the cumulative number of cells infected with each variant in mixed infections, shown as a function of the ratio between the rate of IFN-mediated apoptosis and the rate of IFN-mediated immunization *k*_*A*_/*k*_*R*_ (mean ± SEM values from ten replicate simulations). Left: fitness of W and D relative to pure W infections. Center: direct and indirect fitness components. Right: descriptors of spatial structure.
(TIF)

S1 File A .rar file containing the MATLAB scripts that perform the simulations and associated files. (RAR)
