# Supplementary figures and images for: The role of spatial structure in the evolution of viral innate immunity evasion: A diffusion-reaction cellular automaton model
Source: PLoS Comput Biol. 2020 Feb 10;16(2):e1007656. doi: 10.1371/journal.pcbi.1007656 (PMC7034925; doi:10.1371/journal.pcbi.1007656)

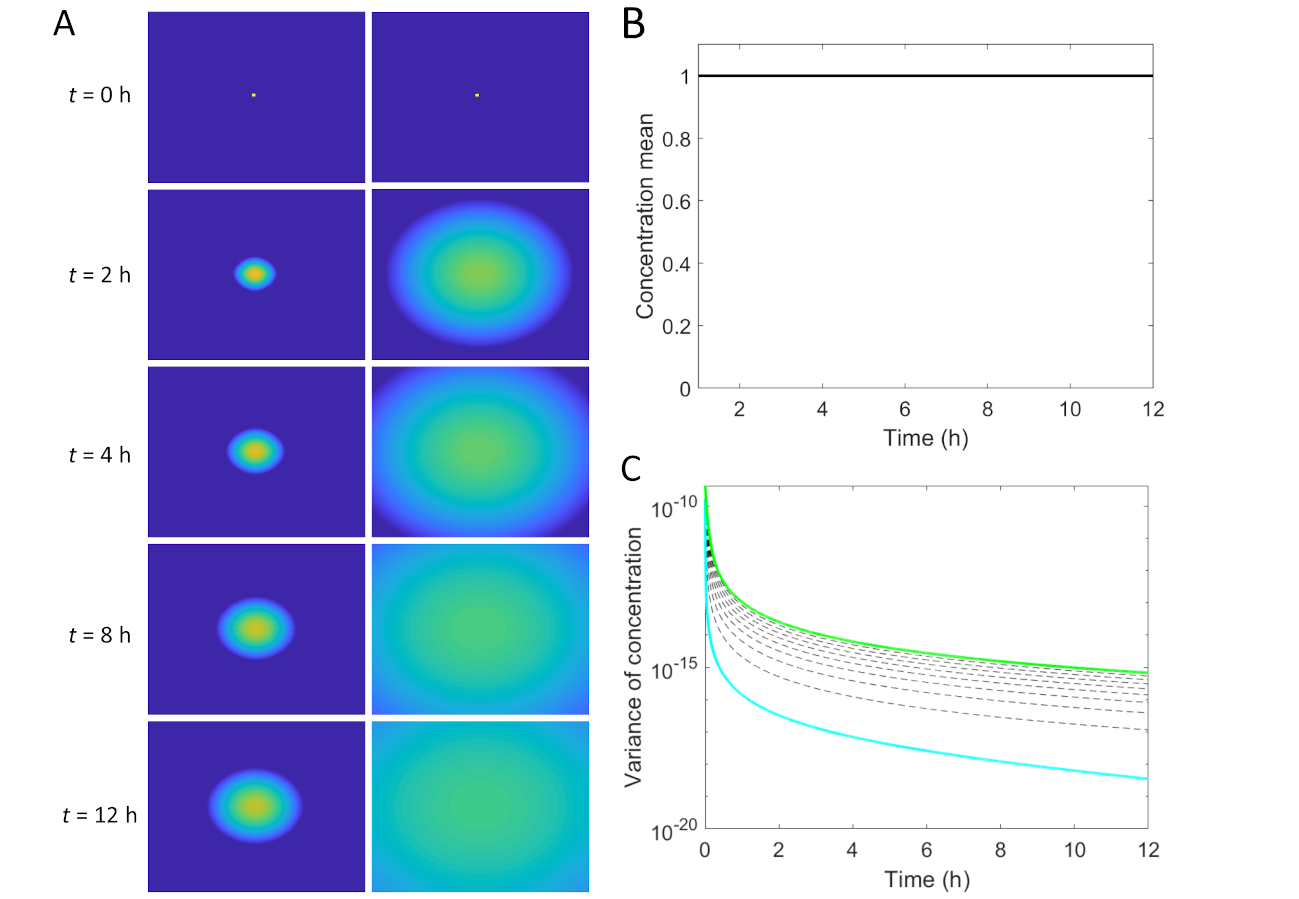

Supplement: S1 Fig — A. Diffusion of particles with a hydrodynamic diameter of 180 nm (virions, left) and 7 nm (IFN, right). B. Mean particle concentration with time (in arbitrary units). C. Variance of the particle concentration with time (green: 180 nm; blue: 7 nm; dashed lines: intermediate sizes). (TIF) [file pcbi.1007656.s001.TIF]

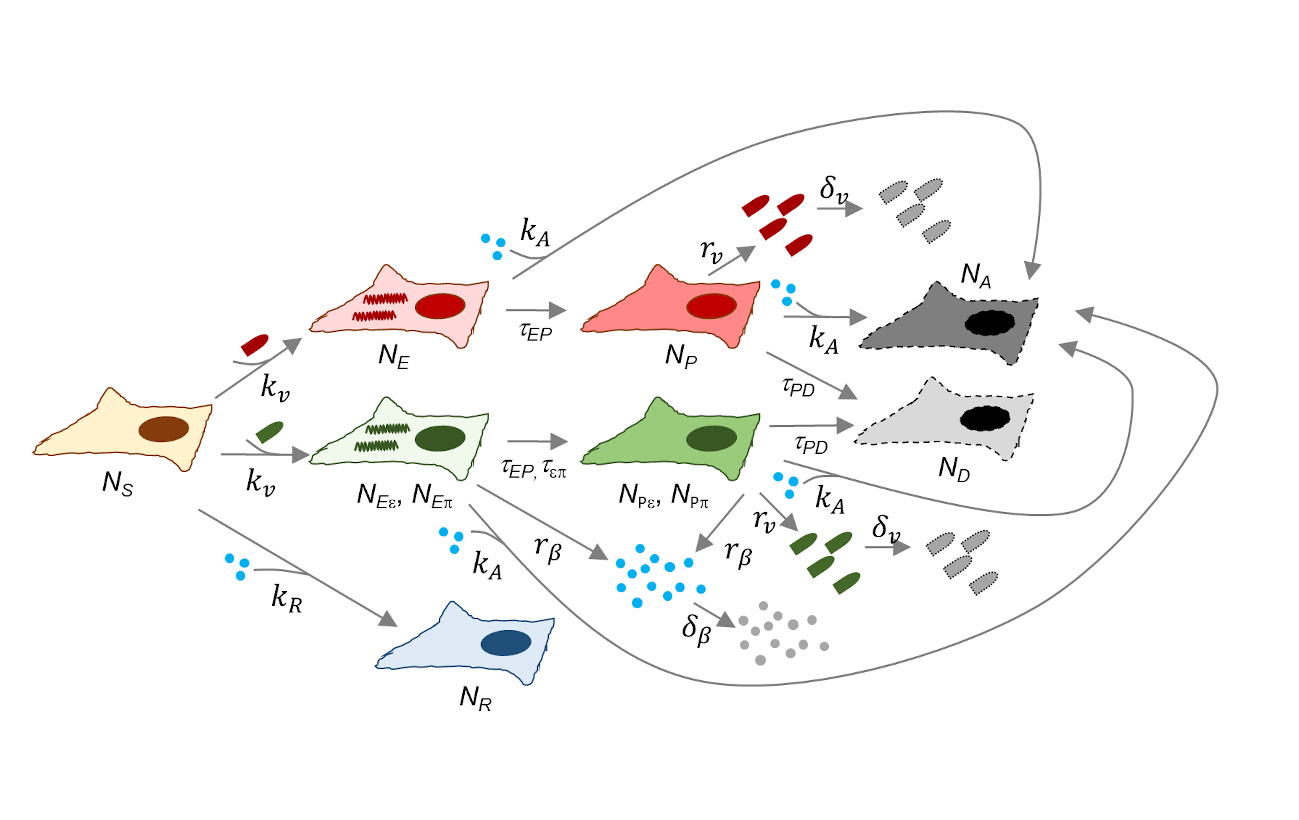

Supplement: S2 Fig — NS: susceptible (non-infected) cells. NE: infected cells in eclipse phase. NP: virion-producing cells. ND: cells killed by the virus. NR: immunized cells (blue). W-infected cells: red. D-infected cells: green. An eclipse phase between viral sensing (Nε) and IFN secretion (Nπ) is also considered. The relative speed of infection and IFN production can vary. Hence, D-infected cells can be in four possible stages (NEε, NPε, NEπ, NPπ). kv: virion infectivity (infection rate). kR: IFN immunization rate. τEP: viral eclipse half time. τεπ: half time between infection and IFN secretion. τPD: half time between virion production and cell death. Thus τPD + τEP, is the total duration of the infection cycle. rv: virion production rate. K = rvτPD is thus the number of virions produced per infected cell. δV: virion degradation/outflow rate. rβ: IFN production rate of immunized cells. δβ: IFN degradation/outflow rate. Infected cells also respond to IFN by undergoing apoptosis (NA cells). kA: IFN-induced apoptosis rate of infected cells. (TIF) [file pcbi.1007656.s002.TIF]

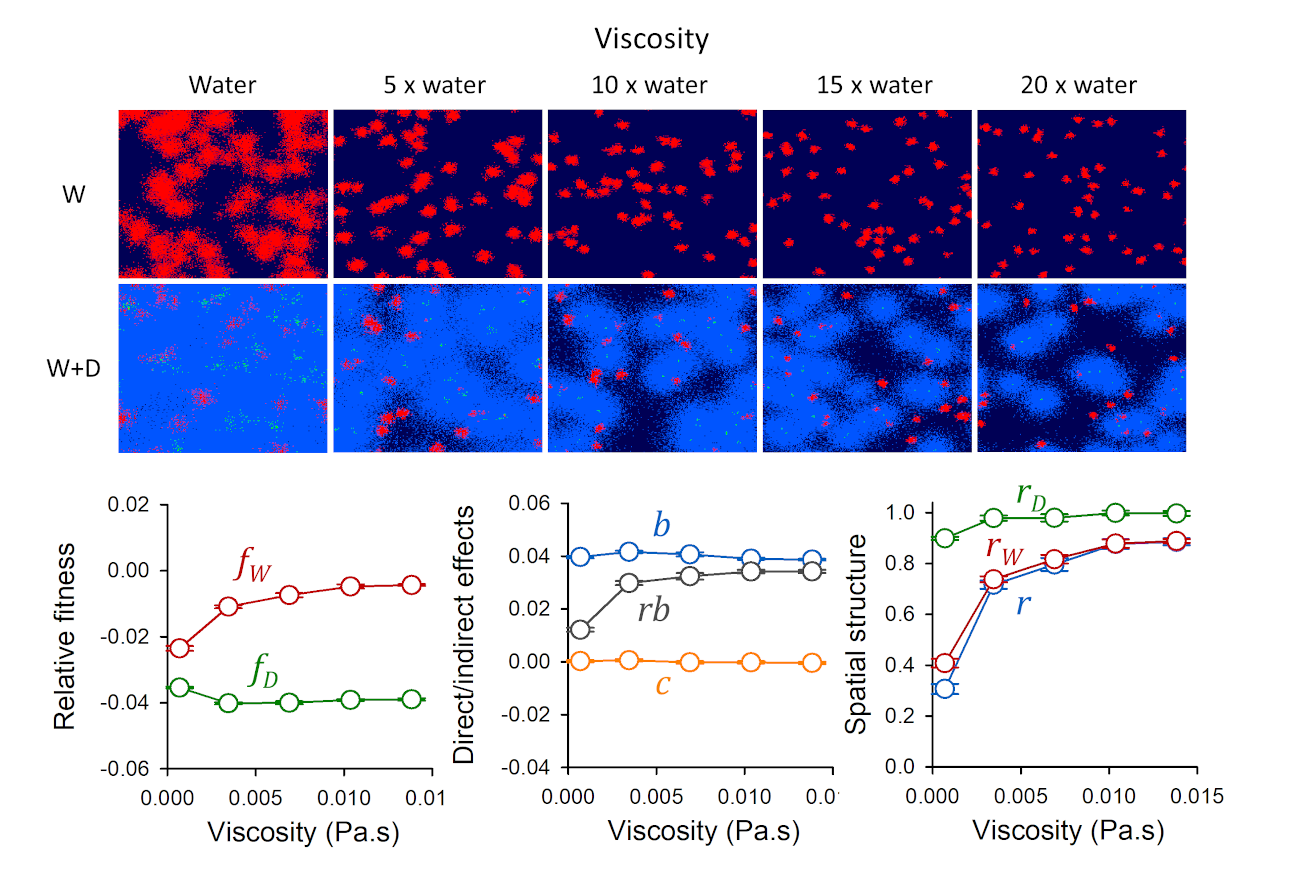

Supplement: S3 Fig — Top: spatial structure of the infection at endpoint (36 h) for increasing viscosities. Pure W infections and mixed (W+D) infections are shown. Susceptible cells are shown in dark blue, cells infected with the W virus in red, cells infected with the D virus in green, and immunized cells in light blue. Infected cells are shown in a cumulative manner, meaning that all cells infected with each variant throughout the progression of the infection are depicted. Thus, dead cells are not shown. Bottom: time-integrated fitness components calculated using the cumulative number of cells infected with each variant in mixed infections, shown as a function of medium viscosity (mean ± SEM values from ten replicate simulations are shown). Left: fitness of W and D relative to pure W infections. Center: direct and indirect fitness components. Right: descriptors of spatial structure. (TIF) [file pcbi.1007656.s003.TIF]

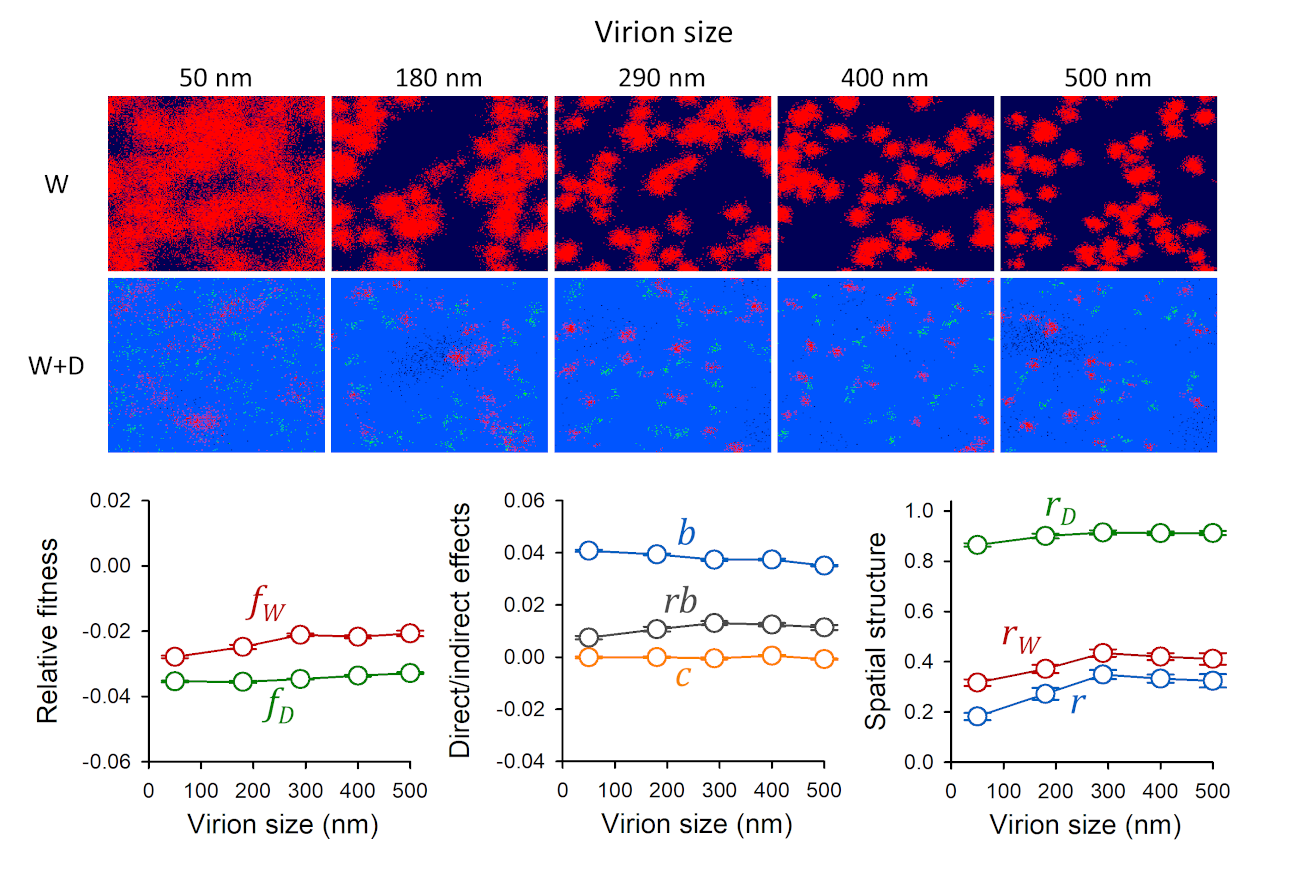

Supplement: S4 Fig — Top: spatial structure of the infection at endpoint (36 h) for increasing virion sizes. Pure W infections and mixed (W+D) infections are shown. Susceptible cells are shown in dark blue, cells infected with the W virus in red, cells infected with the D virus in green, and immunized cells in light blue. Infected cells are shown in a cumulative manner, meaning that all cells infected with each variant throughout the progression of the infection are depicted. Thus, dead cells are not shown. Bottom: time-integrated fitness components calculated using the cumulative number of cells infected with each variant in mixed infections, shown as a function of virion size (mean ± SEM values from ten replicate simulations are shown). Left: fitness of W and D relative to pure W infections. Center: direct and indirect fitness components. Right: descriptors of spatial structure. (TIF) [file pcbi.1007656.s004.TIF]

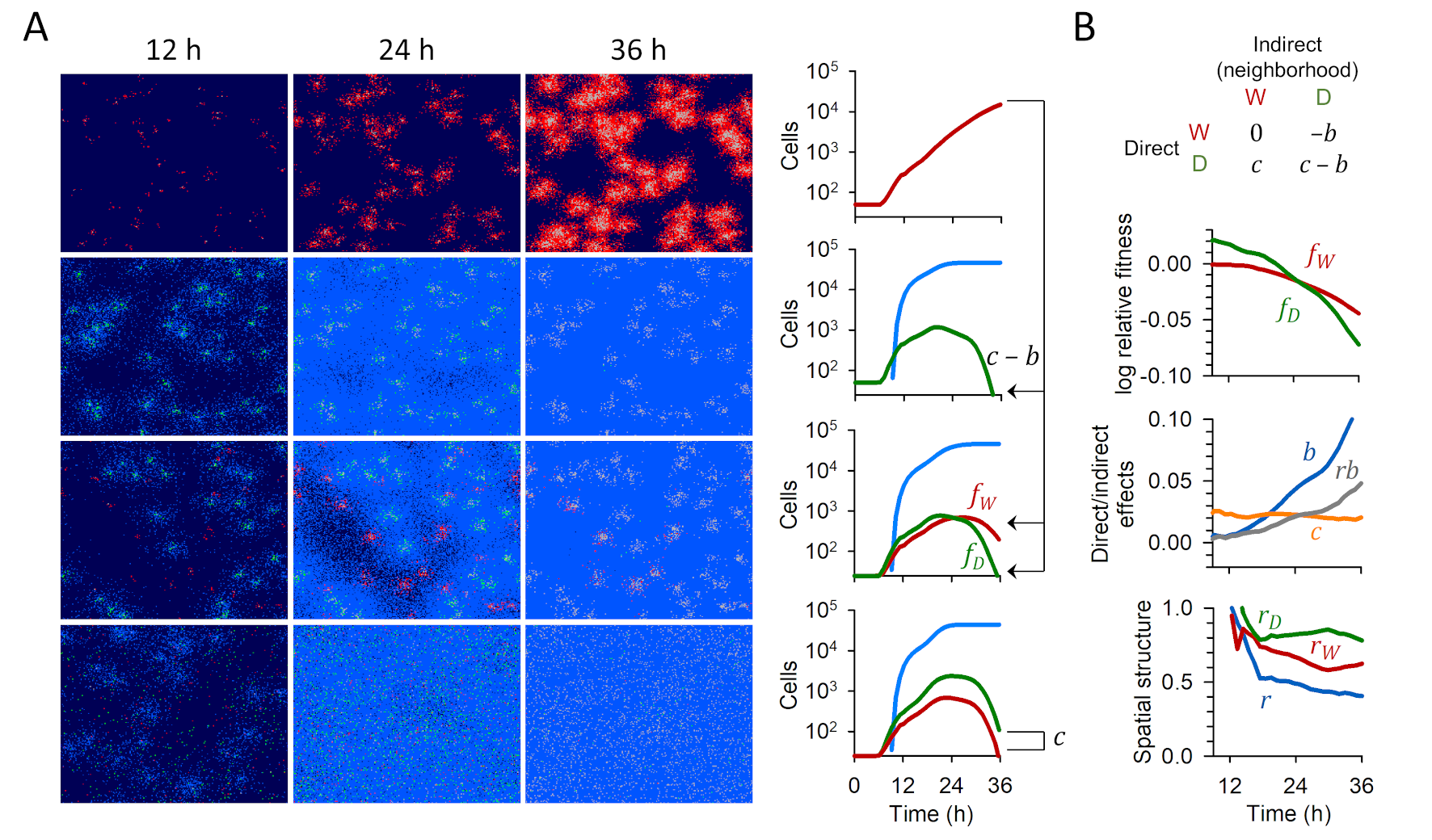

Supplement: S5 Fig — a. Spatial structure of the infection and immune response at different time points (left) and cell counts (right). The color legend is as in Fig 2. Parameter values are as in Table 1, except that virus D produces twice as much progeny virions, i.e. rv(D) = 0.56 virions cell–1 min–1 and rv(W) = 0.28 virions cell–1 min–1. b. Inferred fitness components. The twofold excess progeny produced by D results in a direct fitness advantage c = 0.026 ± 0.001 that counterbalances the indirect fitness advantage obtained by W (see also Fig 4). (TIF) [file pcbi.1007656.s005.TIF]

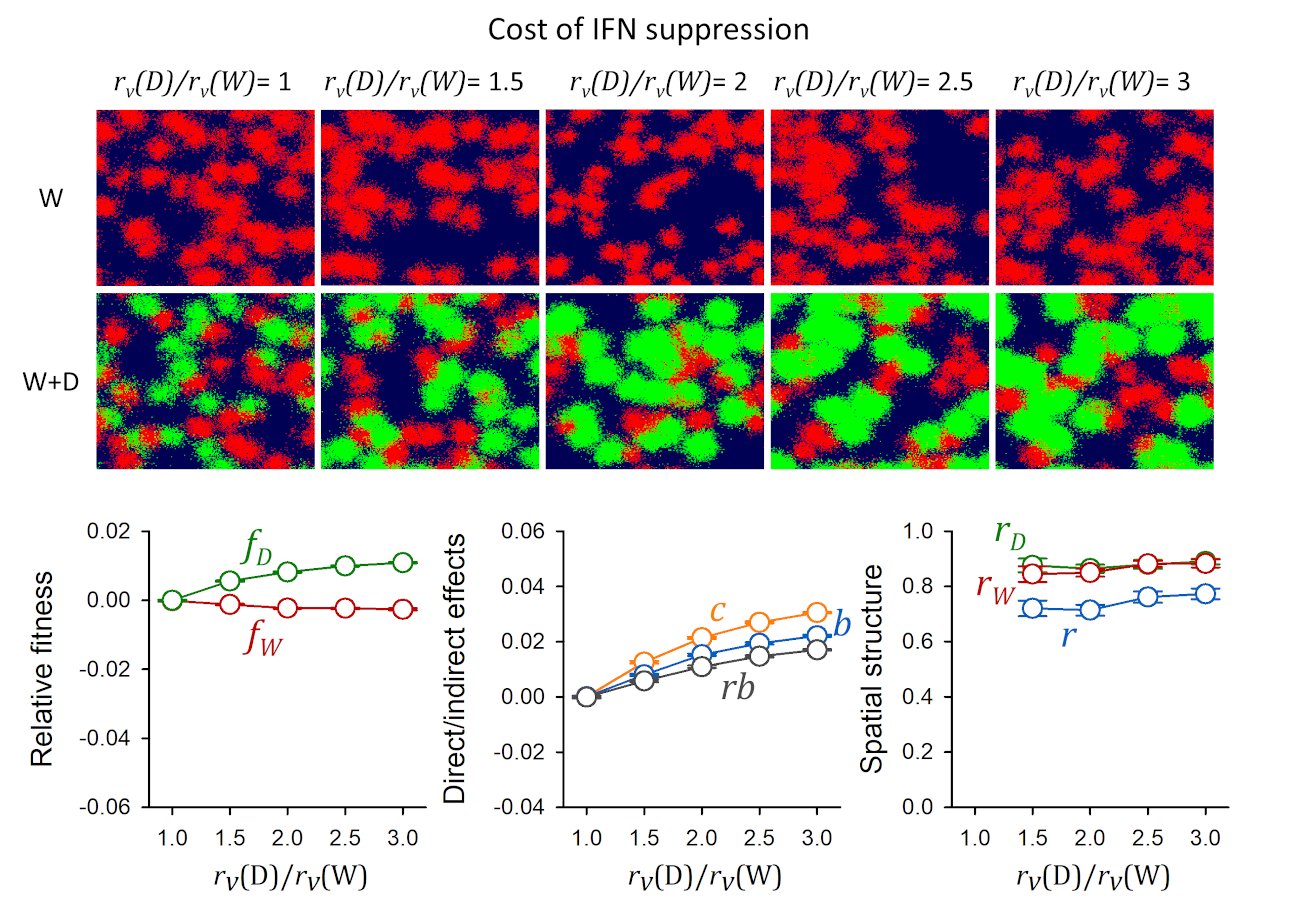

Supplement: S6 Fig — In these simulations, the D virus had a fitness advantage over the W virus in terms of an increased virion production rate, but innate immunity was disabled.Top: spatial structure of the infection at endpoint (36 h) as a function of the cost imposed to the W virus. Only mixed (W+D) infections are shown. Susceptible cells are shown in dark blue, cells infected with the W virus in red, and cells infected with the D virus in green. Infected cells are shown in a cumulative manner, meaning that all cells infected with each variant throughout the progression of the infection are depicted. Thus, dead cells are not shown. Bottom: time-integrated fitness components calculated using the cumulative number of cells infected with each variant in mixed infections, shown as a function of the cost imposed to W (mean ± SEM values from ten replicate simulations). Left: fitness of W and D relative to pure W infections. Center: direct and indirect fitness components. Right: descriptors of spatial structure. (TIF) [file pcbi.1007656.s006.TIF]

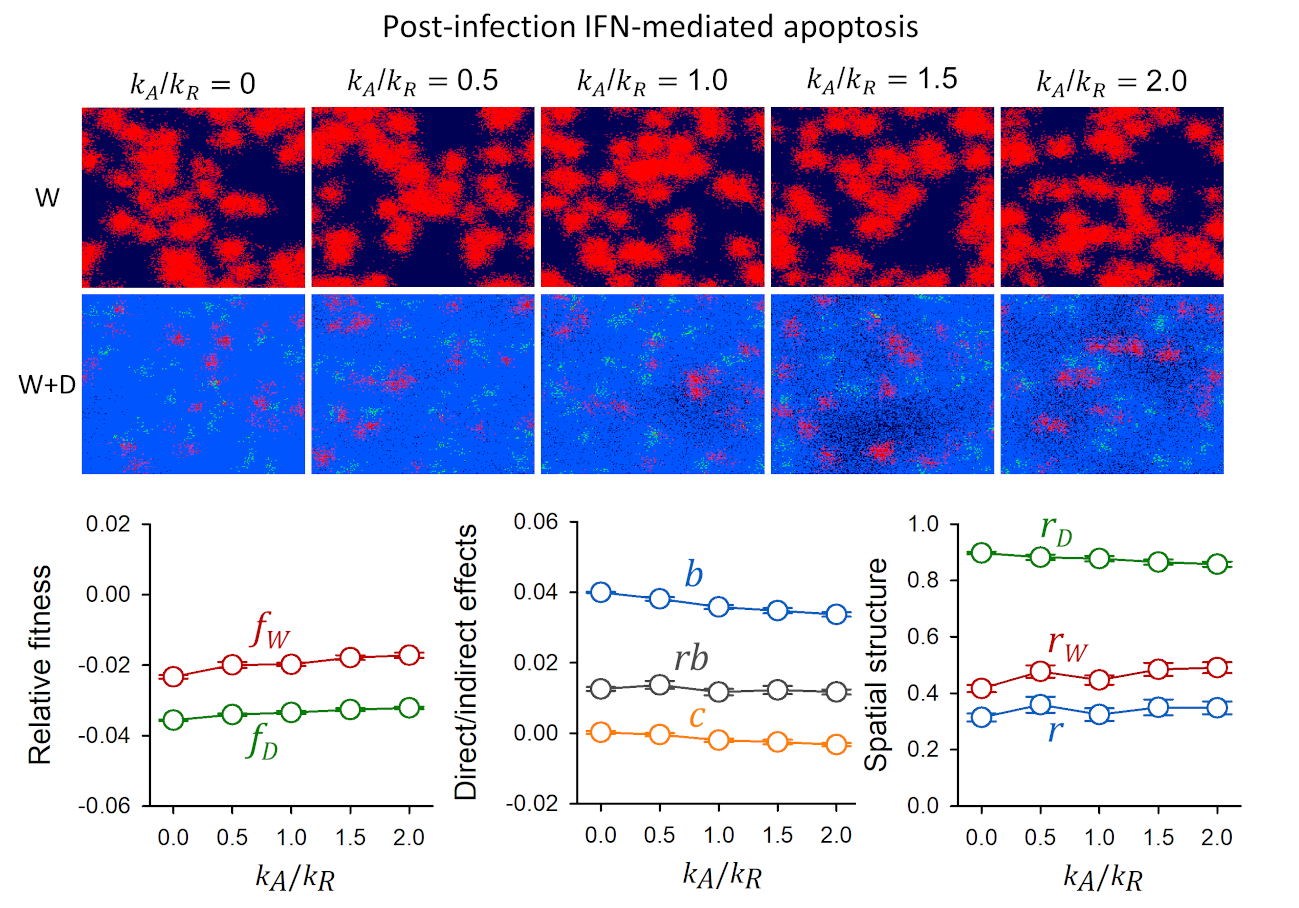

Supplement: S7 Fig — Top: spatial structure of the infection at endpoint (36 h) as a function of the rate of IFN-mediated apoptosis (kA) relative to IFN-mediated immunization (kR). Pure W infections and mixed (W+D) infections are shown. Susceptible cells are shown in dark blue, cells infected with the W virus in red, cells infected with the D virus in green, and immunized cells in light blue. Infected cells are shown in a cumulative manner, meaning that all cells infected with each variant throughout the progression of the infection are depicted. Thus, dead cells are not shown. Bottom: time-integrated fitness components calculated using the cumulative number of cells infected with each variant in mixed infections, shown as a function of the ratio between the rate of IFN-mediated apoptosis and the rate of IFN-mediated immunization kA/kR (mean ± SEM values from ten replicate simulations). Left: fitness of W and D relative to pure W infections. Center: direct and indirect fitness components. Right: descriptors of spatial structure. (TIF) [file pcbi.1007656.s007.TIF]
